# Effectiveness of the computerized balance rehabilitation after hip fracture surgery: A study protocol of a prospective and open-label clinical trial

**DOI:** 10.1097/MD.0000000000012199

**Published:** 2018-09-07

**Authors:** In-Hee Kim, Shi-Uk Lee, Se Hee Jung, Soong Joon Lee, Sang Yoon Lee

**Affiliations:** a Department of Rehabilitation Medicine, Seoul National University Hospital; b Department of Rehabilitation Medicine; c Department of Orthopaedic Surgery, Seoul National University College of Medicine, SMG-SNU Boramae Medical Center, Seoul, Republic of Korea.

**Keywords:** balance, hip fractures, postoperative care, rehabilitation

## Abstract

**Introduction:**

Although balance problems in older populations are directly correlated with hip fractures, the overall physical gain afforded by balance rehabilitation itself has not yet been fully investigated. Here we describe a protocol for an open-label clinical trial to evaluate the effectiveness of computer-based balance-specific exercise (BSE) on the performance and balance of elderly women who underwent hip fracture surgery (HFS).

**Methods and analysis:**

Elderly female patients (≥65 years old) who underwent surgery for femoral neck, intertrochanteric, or subtrochanteric fracture regardless of surgery type will be included. The BSE will be conducted using a computed posturographic system for a 2-week intervention period following HFS. The primary outcome of this study is Berg balance scale score. All functional outcomes will be measured at 1 and 3 weeks and at 3 and 6 months after the surgical intervention. The data will be analyzed using the intention-to-treat principle.

## 1 Introduction

### 1.1 Background

A balance problem is both the cause and the result of hip fracture. In particular, elderly people who have undergone hip fracture surgery (HFS) suffer from balance problems. Radosavljevic et al^[1]^ reported a significant correlation between patient age and Berg balance scale (BBS) score at 3 months after HFS. Also, elderly individuals are afraid of falling after HFS and thus lack both balance confidence and functional balance.^[2]^ Portegijs et al^[3]^ suggested that balance confidence was independently correlated with mobility in patients with fall-related hip fractures. One study also suggested that balance confidence was highly correlated with activities of daily living (ADLs).^[4]^ Therefore, balance impairment may be a long-term issue among older adults after HFS and may limit mobility and daily activities and increase the risk of subsequent falls.^[5]^ Thus, balance impairment should be evaluated as a major fall risk and balance rehabilitation is essential to the prevention of additional falls in elderly individuals after HFS. The American College of Sports Medicine and the American Heart Association have also suggested that community-dwelling older adults at substantial risk of falls should perform exercises that maintain or improve balance to reduce the risk of injury.^[6]^

Most rehabilitation programs after HFS mainly focus on postoperative range of motion exercises, standing, gait training with progressive weight bearing, and strengthening exercises of the hip extensor and abductor muscles. These postoperative programs have been shown to improve the independence of ADLs and gait function after hip fracture.^[7]^ However, in long-term follow-up studies > 1 year, conventional rehabilitation programs had no significant effect on re-fracture and fall rates.^[8]^

Therefore, recent clinical trials about comprehensive rehabilitation after HFS included balance training to strengthen physical functioning and performance with the intention of improving gait and balance.^[9,10]^ One clinical trial also showed that the fall rate was reduced when individualized fall prevention programs were administered to elderly patients who were transferred to the rehabilitation department after HFS.^[11]^ However, those studies were limited to home-based exercises or outpatient rehabilitation programs after general rehabilitation of hip fracture and not about intensive balance training in the early postoperative stage. The overall physical gain by balance rehabilitation itself has not yet been fully investigated. Furthermore, balance training immediately after HFS is fundamentally limited for patients whose balance confidence is low and who still suffer from severe surgical-site pain. Therefore, clinical studies should be conducted to evaluate the effectiveness of systematic and safe balance rehabilitation in patients after hip fracture.

### 1.2 Objectives

We aim to evaluate the effectiveness of a computer-based balance specific exercise (BSE) on the performance and balance ability of the elderly women who underwent HFS. We will also investigate whether the intervention could reduce their fear of falling and improve their coping abilities.

## 2 Method

### 2.1 Trial design

This prospective and open-label clinical trial will be performed in a tertiary hospital setting. During the 2-week postoperative intervention period, patients will participate in the hospital's exercise program starting 5 to 7 days after HFS. All participants will follow the computer-based BSE program. Functional outcomes will be measured at 1 and 3 weeks as well as at 3 and 6 months after surgery. The trial has been registered prospectively with the Clinical Trials.gov Registry (NCT03618576) prior to participant recruitment. Important protocol modifications will be communicated to the trial registry.

### 2.2 Participants and eligibility criteria

Elderly female patients (≥65 years old) who have undergone surgery for femoral neck, intertrochanteric, or subtrochanteric fractures regardless of surgery type (internal fixation, bipolar hemiarthroplasty, or total hip arthroplasty) will be included. Patients who have experienced the following will be excluded: hip surgery for infection, arthritis, implant loosening, or avascular necrosis; femoral shaft fracture, acetabular fracture, isolated fracture of the greater or lesser tuberosity, or periprosthetic fracture; pathologic fracture; combined multiple fracture; revision surgery; severe cognitive dysfunction (obey command ≤ 1 step); cannot stand by supporting a fixed walker at 5 days postoperative; and refusal to participate in a clinical trial.^[12]^

### 2.3 Sample size and recruitment

Because this study is a preliminary and open-label trial, sample size calculation is not needed. A total of 40 subjects will be consecutively recruited. All participants will be enrolled at one tertiary hospital. On the fifth day after HFS, patients who meet the inclusion but not the exclusion criteria will be preliminarily screened by researchers in cooperation with orthopaedic surgeons. Patients who agree to participate in the study will be enrolled.

### 2.4 Intervention

The post-HFS rehabilitation program will be delivered by a rehabilitation physician, physical therapist, occupational therapist, nutritionist, clinical nurse specialist, and social worker. The program consists of total 10 days of physical therapy (PT) sessions (twice per day for 60 minutes) during the 2 weeks after surgery. PT intensity (weight-bearing, strengthening, gait training, aerobic, and functional exercises) will be gradually increased based on the patient's functional level. Intensive patient education will also be provided by multidisciplinary rehabilitation members. BSE will be conducted with a computerized posturographic system for diagnosing balance and movement skills (Balance Master System NeuroCom; Natus Medical Inc., Pleasanton, CA) designed to objectively quantify balance and postural function of different origins, as described previously.^[13]^ Briefly, subjects will be prepared by being introduced to the supported standing position with the objective of looking for a symmetrical load on their legs. In the introductory session, dynamic exercises for small- and medium-sized muscle groups and lower extremity joints will be performed. Movements will be conducted at an average speed with maximum possible amplitudes. After the 5-minute introductory session, subjects will perform the main exercise for 20 minutes while maintaining the tandem position or maintaining their position with and without the use of a proprioceptive bubble. Subjects will be asked to walk on a rectilinear trajectory with or without crutches while changing speed and direction or while performing motor-cognitive tasks such as turning their head to the right and left side following a physiotherapist's input. At the final 5-minute session, the training load will be gradually reduced and conducted at a slow speed and maximum amplitude.

### 2.5 Outcome measures

The following demographic data will be collected at baseline: age, sex, fracture location and laterality, surgery type, and underlying disease. Functional outcomes will be measured at 1 (before intervention) and 3 (after 2 weeks’ intervention) weeks and at 3 and 6 months after surgery. The primary outcome of this study will be balance and fall risk assessed using the BBS^[14]^ (range, 0–56; a lower score indicates a worse outcome). The secondary outcomes will be as follows: physical functioning and walking ability assessed according to the FAC^[15]^ (range, 0–5; a lower score indicates a worse outcome), Koval walking ability scores^[16]^ (range, 1–7; with a higher score indicating a worse outcome), which rate physical functioning according to walking dependency, MRMI^[17]^ (range: 0–40; a lower score indicates a worse outcome), and the MFES^[18]^ (range: 0–140; a lower score indicates a worse outcome); cognition evaluated using the Korean version of MMSE^[19]^ (range: 0–30; a lower score indicates a worse outcome); mood evaluated using the Korean version of GDS^[20]^ (range, 0–30: a lower score indicates a worse outcome); QOL evaluated using the EQ-5D^[21]^ (range: 0–1; a lower score indicates a worse outcome); ADLs determined using the Korean version of MBI^[22]^ (range, 0–100; a lower score indicates a worse outcome) and the Korean version of the Instrumental ADL^[23]^ (range, 0–3; a higher score indicates a worse outcome); and frailty, assessed based on the Korean version of the Fatigue, Resistance, Ambulation, Illnesses, and Loss of weight scale (FRAIL) using the Korean version of the FRAIL scale^[24]^ (range, 0–5; a lower score indicates a worse outcome).

### 2.6 Data analysis

Data will be collected using a standardized data entry form and entered into the data management system. The intention-to-treat principle will be used for the data analysis. Participant characteristics will be described using means and standard deviations for continuous data and frequencies and percentages for categorical data. To compare paired data (before and after) between 2 different points, we will use the paired *t*-test and the Wilcoxon signed-rank test for continuous and nonparametric data, respectively. To compare categorical data, we will use McNemar's test. Statistical significance will be defined as a *P* value < .05. All statistical analyses will be performed using SPSS version 19.0 for Windows (IBM Corp., Chicago, IL).

### 2.7 Ethics and dissemination

The study will be performed according to the relevant guidelines of the Declaration of Helsinki 1964 as amended in Tokyo, 1975; Venice, 1983; Hong Kong, 1989; and Somerset West, 1996.^[25]^ Written informed consent for all interventions and examinations will be obtained at patient admission. The ethics board will be informed of all serious adverse events and any unanticipated adverse effects that occur during the study. The study protocol has been registered at Clinicaltrials.gov and will be updated accordingly. The study methods were designed in accordance with the SPIRIT guidelines for reporting randomized trials.^[26]^ Direct access to the source data will be provided for monitoring, audits, REC/IRB review, and regulatory authority inspections during and after the study. All patient information will be coded anonymously with only the study team having access to the original data. The study results will be disseminated in peer-reviewed publications and conference presentations.

## 3 Discussion

The balance problems of older populations are directly correlated with fragility fractures such as hip fractures that can cause serious morbidity and mortality. Therefore, it is necessary to anticipate and seek to solve such problems. Several types of balance training have been introduced in clinical settings. The main feature of the training is to allow maintenance of body position, both statically and dynamically, over the base of support within defined stability limits.^[27]^ Balance training has historically consisted of walking up and down stairs^[28–30]^ and/or walking on uneven surfaces (a rugged floor or fluffy sponge).^[31]^ Monticone et al^[32]^ suggested that balance task-specific training was superior to general exercises for improving physical function, balance, and ADLs in elderly patients who have undergone internal fixation following hip fracture. A computerized balance-training machine with a force plate that detects weight loads and a monitor giving visual feedback was recently used to train elderly subjects.^[33]^ Dodd et al^[34]^ showed that the training was feasible and useful after HFS in the elderly. However, no clinical trials of computerized balance training in elderly individuals after HFS have been reported to date. Our study will be the first to our knowledge to examine the efficacy and safety of computer-based BSE in this population.

## Author contributions

**Conceptualization:** Shi-Uk Lee, Se Hee Jung, Soong Joon Lee, Sang Yoon Lee.

**Funding acquisition:** Sang Yoon Lee.

**Investigation:** In-Hee Kim, Shi-Uk Lee, Se Hee Jung, Sang Yoon Lee.

**Methodology:** In-Hee Kim, Sang Yoon Lee.

**Project administration:** Shi-Uk Lee, Soong Joon Lee, Sang Yoon Lee.

**Resources:** Soong Joon Lee.

**Supervision:** Sang Yoon Lee.

**Writing – original draft:** In-Hee Kim, Sang Yoon Lee.

**Writing – review & editing:** In-Hee Kim, Shi-Uk Lee, Se Hee Jung, Soong Joon Lee, Sang Yoon Lee.
